# Recent Developments in Sigma-2 Receptor Compounds for Pain

**DOI:** 10.7759/cureus.59882

**Published:** 2024-05-08

**Authors:** Robert B Raffa, Joseph V Pergolizzi

**Affiliations:** 1 Pharmacology and Therapeutics, Temple University, Philadelphia, USA; 2 Pain Management, Nema Research Inc., Naples, USA

**Keywords:** drug discovery, neuropathic pain, analgesia, tmem97, s2r subtype, sigma receptors

## Abstract

After years of enigmatic pharmacology, non-selective ligands, and uncertain clinical application, sigma receptors have emerged as interesting therapeutic drug discovery-development targets. Two subtypes of sigma receptors have now been cloned, sigma-1 receptor (S1R) and sigma-2 receptor (S2R), and there has been much complementary and converging information from advances in molecular biology, computer modeling, virtual screening, and in vitro and in vivo testing. One of several evolving areas of therapeutic potential is for the treatment of pain. In particular, there is accumulating recent evidence from preclinical models that the demonstrated positive effects of S2R compounds in these models suggest possible positive implications for clinical effectiveness against pains that have a neuropathic component. Such pain conditions have imperfect therapeutic options currently. The addition of new drugs to the now available armamentarium would represent a very significant advance for the large number of patients who suffer from these types of intractable pain. Further research is needed to identify and characterize compounds that have not only good in vitro activity but also the characteristics needed to enter clinical trials. Here, we summarize some of the recent reports of the analgesic activity of S2R compounds.

## Introduction and background

The analgesic activity of ligands that are selective for the sigma-2 receptor subtype (S2R) has only relatively recently been studied and reported. The findings of analgesic activity in these non-opioid compounds suggest novel targets for the discovery and development of new drugs for the treatment of pain and, in particular, for the treatment of the serious condition of neuropathic pain. There is a need for better treatment options for neuropathic pain conditions. The positive findings in these studies provide pathways for discovery targets for the development of non-opioid analgesics that could be useful as stand-alone therapeutics or as adjunctive opioid-sparing therapy. Although sigma receptors have a long history of study, early enigma about their structure, function, and pharmacology obfuscated their potential therapeutic utility. The picture is now clearer following the cloning and availability of crystal structures for S1R and S2R. Also new is the recognition of a ligand-sensitive chaperone nature of sigma receptors [1]. This property aids in the proper transport and folding of proteins, a property that is required for proper functioning. Such an activity would have broad therapeutic utility in situations of dysregulated or disrupted physiological pathways or excursions from baseline activity, such as is hypothesized to occur in neuropathic pain [2,3]. Here, we review some recent publications that provide examples of the analgesic activity of some S2R-selective compounds.

## Review

Purpose

In the last few years, selective S2R (agonist) compounds have been reported to display analgesic activity and, due to increasingly specific action at S2R sites vs S1R sites and other receptors, offer promising minimization of off-target adverse effects. We believe it is of value to present a short review of some recent publications that report on the medicinal chemistry and computational techniques used to identify S2R-selective ligands. Where available, we deemed the information on the pharmacologic properties and analgesic activity of these compounds to be of significant potential clinical significance. 

Methods

A literature search for recent publications in the English language was conducted. Using databases such as PubMed, Google Scholar, Ovid MEDLINE®, etc., using Medical Subject Headings (MeSH) search terms such as 'sigma receptor', 'pain', 'analgesia', and combinations of them. Citations within identified publications were searched. In addition, review articles on broad topics were used for background information. The material was reviewed and rated for applicability for the specific target topic, namely, the identification of compounds reported to possess selective S2R activity that, alone or coupled with another analgesic mechanism of activity, related to the potential clinical analgesic activity.

Results

S2R-induced Antinociception (Analgesia)

To our knowledge, the first report that the S2R could be a possible pain target was published in 2017 [4]. We include this and some additional examples that support this important potential clinical application since additional options for neuropathic pain would represent a significant advancement. The preclinical data are summarized, not the absorption, distribution, metabolism, elimination (ADME) and toxicology, because the emphasis is on proof-of-principle more than whether or not an individual compound has the requisite druggability properties (e.g., pharmacokinetics, etc.) that would advance it into clinical trials.

The S2R had only recently been cloned and recognized to be the same as transmembrane protein 97 (Tmem97) [5] when Sahn et al. in 2017 [4] found that chlorinated norbenzomorphans and methanobenzazocine structures provided good chemical scaffolds as starting points to prepare compounds with binding affinity for S2R [6]. The potential antineuropathic pain activity of the compounds was assessed in adult male mice (C57Bl6/J) using the standard preclinical spared nerve injury (SNI) surgery model [7]. This partial denervation procedure consists of ligation-induced injury to the common peroneal and tibial nerves, leaving the sural nerve intact. This results in hypersensitivity to touch in the region of skin innervated by the spared sural nerve on the same side as the surgery. Skin sensitivity remains normal on the side contralateral to the site of injury. The ipsilateral hypersensitivity develops over about a week and is maintained for the subsequent week. Hypersensitivity is assessed as the decrease in paw-withdrawal threshold to an applied mechanical stimulus applied to the hindpaw of the mouse using calibrated von Frey filaments.

The tested compounds were administered intrathecally (IT) into the intervertebral space between L5 and L6. Several mixed S1R/S2R compounds were tested; the more S2R-selective ones are highlighted here. As previously reported, several S1R compounds, namely JWG-1014, JSS-1027, and MFG-1046, produced anti-mechanical-hypersensitivity effects by 48 hours post-IT injection that persisted for at least 72 hours after the injection. Two compounds with significantly greater affinity selectivity for S2R than S1R (ratio of nM *K*_i_ values) were tested: UKH-1114, selectivity = 1,279/46 = 28, and siramesine (Lu-28-179), which labels an S2R site in human brain [8] with selectivity = 79/0.12 = 658. Of note, the high selectivity of siramesine would be expected to translate into clinically meaningful clinical separation. Based on the favorable physicochemical properties of UKH-1114, it was also injected intravenously (IV) in the same neuropathic pain model. At 10 mg/kg IV, UKH-1114 produced essentially complete elimination of mechanical hypersensitivity at 48 hours after infusion (onset at three hours and return to vehicle baseline by 72 hours). For comparison, gabapentin produced essentially complete elimination of hypersensitivity at one and three hours after infusion at 100 mg/kg IV. Importantly, since UKH-1114 is inactive at a panel of other receptor sites, and the modest effect of IT UKH-1114 was completely blocked by the S2R agonist SAS-0132, the antineuropathic pain effect seems likely due to its S2R agonist action.

Although not for the express purpose of identifying analgesic agents, Floresta et al. [9] nevertheless described the use of a structure- and ligand-based virtual screening method to identify potential S2R ligands in a chemical library of small molecule natural products. The source of the 1517-compound library was the union of the Seaweed Metabolite database and the Chemical Entities of Biological Interest database. The 2D structures that were so obtained underwent molecular mechanics energy minimization, with the protonation states calculated for a pH of 7.0. The 3D structures were developed using 3D quantitative structure-activity relationship (3D-QSAR) statistical models. The resultant 3D structures were docked in silico to a S2R homology model based on the sequence of the human S2R. Of the original 1517 compounds, 42 were predicted to have sufficient affinity for S2R (*K*_i_ values ≤100 nM) to be of interest (the Ki values ranged from 10 nM to 0.01 nM). The highest mean affinity in the combined filters was 0.6 nM. None of the compounds were tested for in vivo activity, but the technique provides a template for evaluating chemical libraries for S2R-targeted compounds. Interestingly, one of the compounds that was described possesses a chemical structure that is similar to progesterone, which has been linked to sex differences in pain and analgesic response [10].

By 2020, the McCurdy group had been working on developing selective S2R compounds but not concentrating on pain. Motivated by the report by Sahn et al. [4] on the anti-neuropathic pain effect of S2R agonists, Intagliata et al. [11] reported the discovery and testing of CM398 1-(4-(6,7-Dimethoxy-3,4-dyhydroisoquinolin-2(1H)-yl)butyl)-3-methyl-1H-benzo(d)imidazol-2(3H)-one. The motivation was an appreciation from structure-activity relationships (SARs) that the combination of the benzimidazolone as a scaffold and the 6,7-disubstituted tetrahydroisoquinoline as a cyclic amine fragment provided a likely basis for obtaining an opportunity to improve upon previous compounds possessing S2R affinity. This was accomplished by a nearly six-fold increase in S2R binding affinity and more than at least a five-fold increase in selectivity over S1R.

Compound CME398 was found to have a subnanomolar *K*_i_ value at S1R (rat brain) of 0.43 nM and about 1.300-fold selectivity over S1R (*K*_i _= 560 nM) - believed to be the largest separation reported up to that time - and at least 10-fold selectivity over neurotransmitter receptors (dopamine, serotonin, opioid, and N-methyl-D-aspartate (NMDA)) and transporters (dopamine, serotonin, and norepinephrine). CME398 was tested for antinociceptive effects using the formalin-induced inflammation test in mice (10-week-old male CD-1). CME398 was administered intraperitoneally (IP) 10 minutes before formalin injection into the hind paw. CME398 produced a dose-related (0.23 to 23.2 mg/kg) inhibition of formalin-induced hind paw licking up to nearly 100% inhibition. 

S2R-induced Vs S1R-induced Antinociception (Analgesia)

It is appropriate at this juncture to ask the question: given the extensive literature on S1R-induced antinociception in a variety of acute and neuropathic pain models, how is the same effect produced by the other sigma receptor subtype S2R?

An answer to this question has been provided by Sánchez-Blázquez et al. [12]. They first review the development of knowledge about S1R and S2R. The S1R was purified from pig brain, sequenced, and cloned in 1996 [13]. It was found to be a 25.3 kDa protein, distinct among receptors, bearing little sequence homology to any other mammalian receptor. The human S1R was characterized 20 years later using complementary X-ray crystallography combined with fitting known S1R ligands in complex with the proposed S1R structure [14]. S1R knockout (KO) mice (S1R^-/-^) do not develop neuropathic pain-like allodynia in several different animal models [15-18]. Multiple studies have reported the antinociceptive and/or antineuropathic pain effects produced by S1R antagonists administered alone, together with other analgesics, or as single 'bispecific' compounds having dual S1R-antagonist plus other analgesic mechanisms of action in a single molecule (submitted for publication). The cloning of the S2R occurred much later than S1R, ending a decades-long search [19]. It is distinct from S1R. Sánchez-Blázquez et al. [12] then summarize the evidence for S2R-agonist mediated antinociception, principally that S2R selective compounds reduce mechanical hypersensitivity in the spared nerve injury model, as described above. Synthesizing these results, the authors conclude that both S2R activation by agonists and S1R inhibition by antagonists promote comparable anti-neuropathic pain effects, which they point out suggests that both subtypes of SRs are involved in regulating neuropathic pain pathways, but in opposite directions, yet in some coordinated or coupled manner. 

Sánchez-Blázquez et al. [12] postulate a reciprocal relationship in pain control between S1R and S2R, namely that S2R facilitates and S1R inhibits endogenous (opioid) pain pathways and that the subtypes exchange roles during the occurrence of neuropathic pain perception. They propose that the S2R is involved in the transmission of the analgesic effects of agonists at the mu-opioid receptor (MOR), including the opiate morphine and the endogenous opioid ß-endorphin and the synthetic opioid (D-Ala2, N-MePhe4, Gly-ol)-enkephalin (DAMGO). Among the evidence supportive of their view a) S2R KO mice develop mechanical allodynia following nerve constriction injury-induced neuropathic pain (measured as an enhanced hind paw withdrawal response to von Frey filaments), whereas S1R deficient mice do not; 2) the hypersensitivity that developed in the S2R KO mice was alleviated by the S1R antagonist S1RA, and 3) there was no difference in response between S2R vs wild-type (WT) mice to non-MOR compounds (such as a delta-opioid agonist, cannabinoid agonist, or alpha-2 adrenoceptor agonist). Taken together, the evidence provides a mechanistic explanation of how S2R agonists, as well as S1R antagonists, can produce analgesic effects in models of acute and neuropathic pain.

The Future Direction of S2R Analgesic Drug Discovery

S2R is now a relatively well-characterized receptor, and it has now been identified as a potential therapeutic target for the treatment of a variety of clinical pain conditions, including conditions with a component of neuropathic pain. Some interesting recent examples of the way forward are summarized below. 

**Figure 1:**
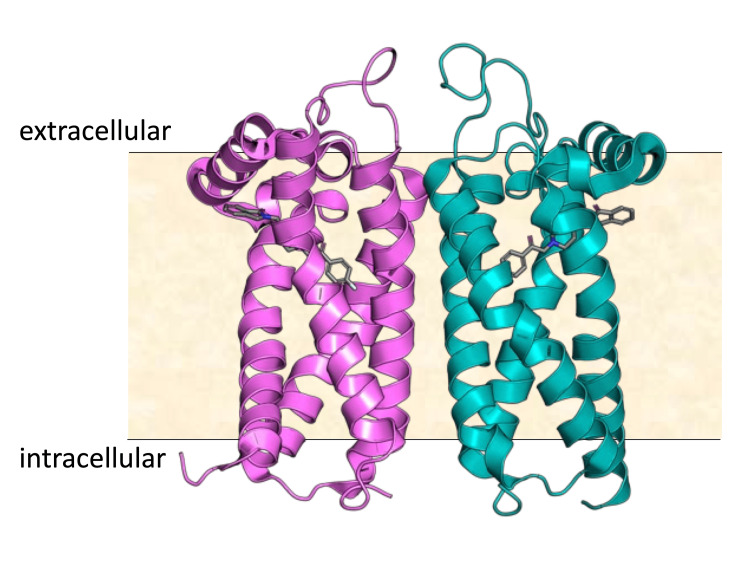
Bovine sigma-2 receptor bound to roluperidone Image modified with permission [21]

Alon et al. [20] report a tour-de-force screening of 490 million virtual molecules in which they employed a combination of a biochemical and structural approach in conjunction with computational docking against the S2R, with a comparison with the roluperidone-binding pose (Figure 1) [21]. A ligand-binding cavity is created near the center of the S2R by 'kinks' formed by the presence of proline amino acids in each of the four transmembrane helices of S2R. The binding cavity, which is lined with lipophilic and aromatic residues, opens laterally into the lipid membrane bilayer. Thus, ligands enter in their neutral deprotonated form and then become protonated within the binding site, where they form a salt bridge with Asp29. A subset of 484 compounds of the original 490 million compounds were synthesized and further screened. Through the process of sequential screening, 127 compounds were identified that have affinities for S2R of greater than 1 µM, then 31 compounds were identified that have affinities for S2R greater than 50 nM, then three compounds were identified with affinity for S2R of 3-48 nM. Importantly, the final three had selectivity of S2R over S1R of up to 250-fold.

The final three compounds resulting from the above screening (Z1665845742, Z4857158944, and Z4446724338) were tested for analgesic effects using the SNI model of neuropathic pain in mice (ligation and transection distally of two of the three branches of the sciatic nerve, leaving the sural nerve intact). Seven to 14 days following surgery, the mice were tested for hind paw mechanical hypersensitivity to von Frey filaments (anti-allodynia indication). The mice received Z1665845742 (S2R *K*_i_ = 4.6 nM, S2R-selective,), Z4857158944 (S2R Ki = 4 nM, S2R-selective), or Z4446724338 (non-selective S2R/S1R) subcutaneously 30-min prior to testing. Both of the S2R-selective compounds, Z1665845742 and Z4857158944, increased hind paw withdrawal latency in the mouse SNI model, suggestive of an anti-allodynic effect. The onset of effect was slower than for the non-selective compound Z4446724338 but was the same by 24 hours. Since the delay cannot be easily explained by the pharmacokinetic properties of the compounds, the authors speculate that it might reflect longer-term signaling or regulatory (chaperone) effects. The authors also explored whether analgesic tolerance would develop during four daily injections. The antinociceptive effect of Z4857158944 was lost by the third injection, indicating that development of tolerance to the analgesic effect had developed. 

The same group recently provided additional evidence that the S2R is a promising target for the treatment of conditions of neuropathic pain [22]. They demonstrated that the antinociceptive (anti-allodynic) effect of the relatively selective (10-fold) S2R vs S1R compound FEM-1689 (*K*_i_ = 17 nM) is absent in S2R KO mice (male and female, IV administration) in the SNI model, showing the requisite nature of S2R in the anti-allodynic effect. S2R mRNA expression in human lumbar dorsal root ganglia (DRG) was revealed with in situ hybridization using tissue obtained from human donors (one male, two females). Nearly all (>99%) of DRG neurons expressed S2R mRNA S2R, and it was found to be expressed across all sensory neuron subtypes, including voltage-gated sodium channel (Nav) 1.8-positive nociceptors (all expressed S2R), low-threshold mechanoreceptors, and proprioceptors, with particularly high expression in proenkephalin-positive nociceptors and A(delta) low-threshold mechanoreceptors. It was also shown that FEM-1689 inhibits the integrated stress response (ISR) [23] in human DRG neurons in an S2R-dependent manner, providing possible insight into some details of the S2R-mediated analgesic mechanism of action. 

Lu et al. [24] sought to find novel S2R ligands by further exploring the structure-affinity relationships (SAfiR) of analogs, including enantiomorphic compounds, of the piperazine-substituted norbenzomorphan S2R ligands SAS-0132 and DKR-1677. Computational docking gave further support for the importance of salt bridges of S2R ligands with Asp29 and cation-π interactions with Tyr150 to S2R binding affinity and suggests a possible deeper pocket-site interaction with Glu73. The novel compounds had good S2R binding affinity (nM range) but less than 25-fold selectivity over S1R. No in vivo data were presented. 

**Figure 2:**
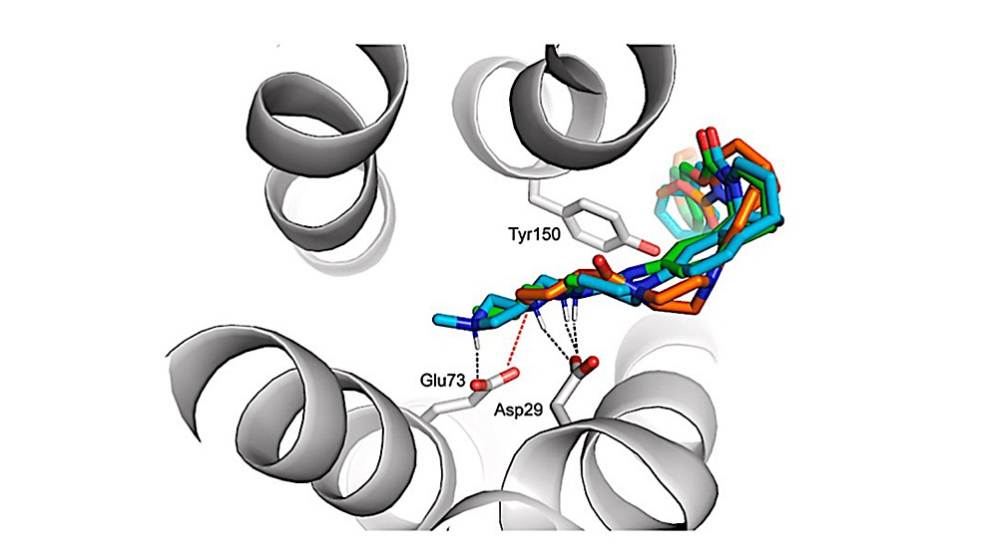
Model of binding pocket of S2R showing important residues for binding Image from Walby et al. with permission [25]

As a final example, Walby et al. [25] very recently explored the structure-activity relationships of novel benzoxazocine, benzomorphan, and methanobenzazepine ligands for S2R activity. Computational docking studies again supported electrostatic interactions (H-bonding with Asp29 and cation-π with Tyr150) with the binding pocket of the S2R (Figure 2). The chemical spaces that were explored yielded compounds of modest affinity for S2R and modest selectivity over S1R, but the process highlights the tremendous advances in S2R drug discovery in the last several years.

## Conclusions

Recent publications have identified several highly selective S2R ligands and assessed their analgesic potential. Perhaps the most important potential impact of these advances is the reported activity of the compounds in animal models believed to be predictive of clinical utility against pains that have a neuropathic pain component. Such compounds would fill an important medical need. In addition, having non-opioid analgesic options, such as S2R-selective compounds, would be an added advantage. Future directions will likely include attempts to improve S2R vs S1R selectivity, or demonstration that some mixture of S1R (agonist or antagonist) with S2R (agonist or antagonist) activity yields good ratios of efficacy and safety; and efforts to discover compounds that possess the ADME, pharmacokinetic, and toxicologic characteristics needed for entry into clinical trials.
